# Metachronous spinal cord involvement B cell and subcutaneous tissue involvement NK/T cell lymphoid proliferations and lymphomas arising in post-transplantation mimicking general NK/T cell lymphoma: a case report and review of the literature

**DOI:** 10.3389/fimmu.2024.1467506

**Published:** 2024-10-14

**Authors:** Yingxin Zhu, Lingbo He, Heshan Zou, Shuyan Yao, Jinglin Hu, Jing Guo, Yini Wang

**Affiliations:** ^1^ Department of Hematology, Beijing Anzhen Hospital, Capital Medical University, Beijing, China; ^2^ Department of General Practice, Beijing Friendship Hospital, Capital Medical University, Beijing, China; ^3^ Department of Hematology, Beijing Friendship Hospital, Capital Medical University, Beijing, China

**Keywords:** transplantation, Nk/T cell lymphoma, lymphoid proliferations and lymphomas associated with immune deficiency/dysregulation, spinal cord, subcutaneous

## Abstract

Lymphoid proliferations and lymphomas arising in post-transplantation are potentially life-threatening complications after solid organ transplant (SOT) and hematopoietic stem cell transplant (HSCT). The lymphoid proliferations and lymphomas arising in post-transplantation originating from different cell lineages in the same patient are highly unusual. Herein, we delineate a case of isolated spinal cord involvement with B cell lymphoid proliferations and lymphomas arising in post-transplantation at 11 months post-transplantation, which was successfully treated with chemotherapy and intrathecal injection. Six months later, the patient again developed lymphoma arising in post-transplantation, presenting with predominant subcutaneous tissue involvement deriving from EBV-positive NK/T cells, and received four courses of chemotherapy. Ultimately, she achieved complete remission (CR). The report further contributes to our new insights into the unusual clinical presentations of lymphoid proliferations and lymphomas arising in post-transplantation.

## Introduction

Lymphoid proliferations and lymphomas arising in post-transplantation used to be termed post-transplant lymphoproliferative disorders, a heterogeneous group of lymphoid and plasmacytic proliferations, which are categorized as “Lymphoid proliferations and lymphomas associated with immune deficiency/dysregulation (IDD)” in the 5th edition of the World Health Organization Classification of hematolymphoid tumors (1). It encompasses a spectrum of disorders ranging from indolent reactive lesions to malignant and aggressive diseases (2). For patients with lymphomas arising in post-transplantation, failure to receive timely and appropriate treatment will result in a 3-year overall survival rate of 20% (3). In comparison, the 3-year overall survival rate significantly improves to 60% when patients receive prompt diagnosis and appropriate management (4). The manifestation of lymphoid proliferations and lymphomas arising in post-transplantation is nonspecific, including fever, night sweats, fatigue, loss of appetite, lymphadenopathy, and enlarging masses, and some patients are asymptomatic, which poses challenges for early diagnosis. Approximately 90-95% of lymphoid proliferations and lymphomas arising in post-transplantation display B cell lineage derivation (5), with a high incidence of extranodal involvement, including frequently the gastrointestinal tract, lung, and bone marrow (6). In contrast, NK/T cell lymphoid proliferations and lymphomas arising in post-transplantation are uncommon. Here, we report a rare case of isolated spinal cord involvement with B cell lymphoid proliferations and lymphomas arising in post-transplantation at 11 months post-transplantation. Six months later, the patient again developed lymphoma arising in post-transplantation, presenting with predominant subcutaneous tissue involvement deriving from EBV-positive NK/T cells.

## Case presentation

A 29-year-old woman presented to an outside hospital with a prolonged fever (>38.5°C). Although receiving antibiotic treatment with meropenem (500mg q8h for 7 days) combined with dexamethasone (5mg qd for 2 days) and Tylenol (0.65g q8h for 7 days), her body temperature was repeatedly elevated. The in-patient examination indicated that sCD25(18964pg/ml), hypertriglyceridemia(3.28mmol/l), hypofibrinogen(0.81g/l), hepatosplenomegaly and hemophagocytosis in the bone marrow. She presented with EBV DNA positivity of plasma and Peripheral Blood Mononuclear Cells(PBMCs), accompanied by bilateral lymph node enlargement in the neck and inguinal areas, and was diagnosed with EBV-HLH according to HLH-2004 diagnostic criteria in November 2019. Subsequently, she was initially treated with the DEP chemotherapy regimen, which consisted of etoposide (110mg day1), doxorubicin hydrochloride liposomes (40mg day1), methylprednisolone (80mg, day1 to 3, 30mg, day4 to 7, 10mg, day8 to 10, and 4mg, day11 to 14) on November 28, 2019 and achieved CR after two cycles of induction therapy according to efficacy evaluation criteria of the HLH (7).

On December 20, 2019, the patient was admitted to our hospital for HSCT. She had no other medical history or family history of primary immunodeficiency. On physical examination, the patient was afebrile, with normal vital signs. The neck, axilla, and groin ultrasound detected no lymph node enlargement. On the functional examination of NK cells and cytotoxic T lymphocytes (CTL), the expression of associated proteins, such as ΔCD107a, perforin, and Granzyme, is normal. Whole exome sequencing (WES) did not also detect any significant pathogenic variant.

On February 20, 2020, she received allogeneic HSCT from her father following a conditioning regimen including busulfan, cyclophosphamide, etoposide, and anti-thymocyte globulin(ATG). Prior to HSCT treatment, the serologic workup of the recipient was positive for EBV and negative for CMV, whereas the donor was serologically negative for EBV and CMV. Cyclosporin A (CsA, 50mg intravenously daily) and mycophenolate mofetil (MMF, 500mg orally daily) were used as prophylaxis against graft versus host disease (GVHD). Grade II hyperacute GVHD of the gastrointestinal tract occurred 4 days after HSCT and was in remission after short course of methotrexate (24mg/day, +4 day, 16mg/day, +6day, and 18mg/day, +9 day), methylprednisolone (40mg qd), cyclosporinA (CsA, 100mg intravenously twice daily), and mycophenolate mofetil (MMF, 500mg orally twice daily) therapy. At 3 months after transplantation, the immunosuppressive therapy regimen was adjusted, consisting of CsA (50mg orally twice daily initially, dosage adjusted according to drug concentration), methylprednisolone (8mg once daily) and MMF (500mg orally twice daily). The immunosuppressive treatment was gradually reduced and discontinued 1 year after transplantation. The patient had been maintaining complete donor chimerism since 20 days after transplantation.

**Figure 1:**
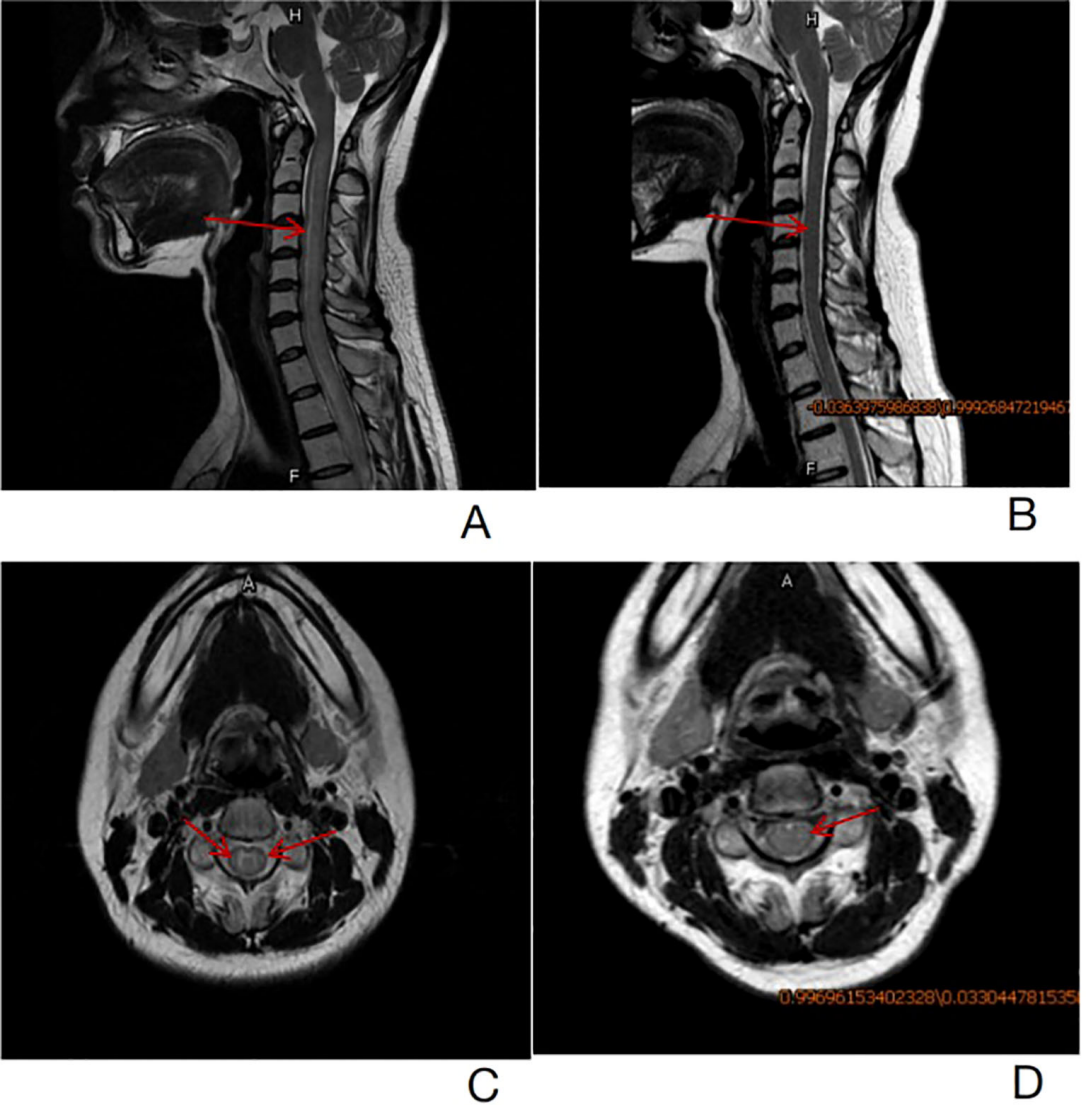
**(A)**-T2 weighted sagittal images revealed diffuse swelling and increased spinal cord signal intensity from cervical 2 to thoracic 3 before treatment (the red arrow). **(B)**- T2 weighted sagittal images showed that spinal cord swelling and abnormal strengthening signals from cervical 2 to thoracic 3 were significantly remitted after treatment (the red arrow). **(C)**- T2 weighted axial images revealed hyper-intense signal in the spinal cord more in the white matter region before treatment (the red arrow). **(D)**- T2 weighted axial images revealed hyper-intense signal in the white matter of the spinal cord return to normal (the red arrow).

At 11 months post-transplantation, the patient was admitted to our hospital with posterior neck pain and limb numbness for 2 months. Magnetic resonance imaging (MRI) of the spine revealed diffuse swelling and increased signal intensity of the spinal cord extending from cervical 2 to thoracic 3. DNA copy numbers of EBV-DNA in both plasma, PBMC and cerebrospinal fluid (CSF) measured by real-time qPCR were positive. The sorting of EBV-infected peripheral blood cells revealed a predominance of B lymphocytes. However, bone marrow and CSF cytology demonstrated no abnormal cells. We eliminated other etiologies such as ischemic myelopathy, compressive myelopathy, autoimmune/infectious/parainfectious myelitis and metabolic/toxic myelopathy by a comprehensive analysis of clinical and MRI findings. Eventually, the patient was clinically diagnosed with EBV-positive lymphoid proliferations and lymphomas arising in post-transplantation with spinal cord involvement according to National Comprehensive Cancer Network (NCCN) guidelines. Consequently, the patient received four courses of chemotherapy treatment that incorporated Rituximab, Reduction in immunosuppression (RIS) combined with intrathecal injection of methotrexate (MTX) and dexamethasone. Her symptoms and spinal cord swelling gradually remitted and nearly completely disappeared (**Figure 1**).

**Figure 2:**
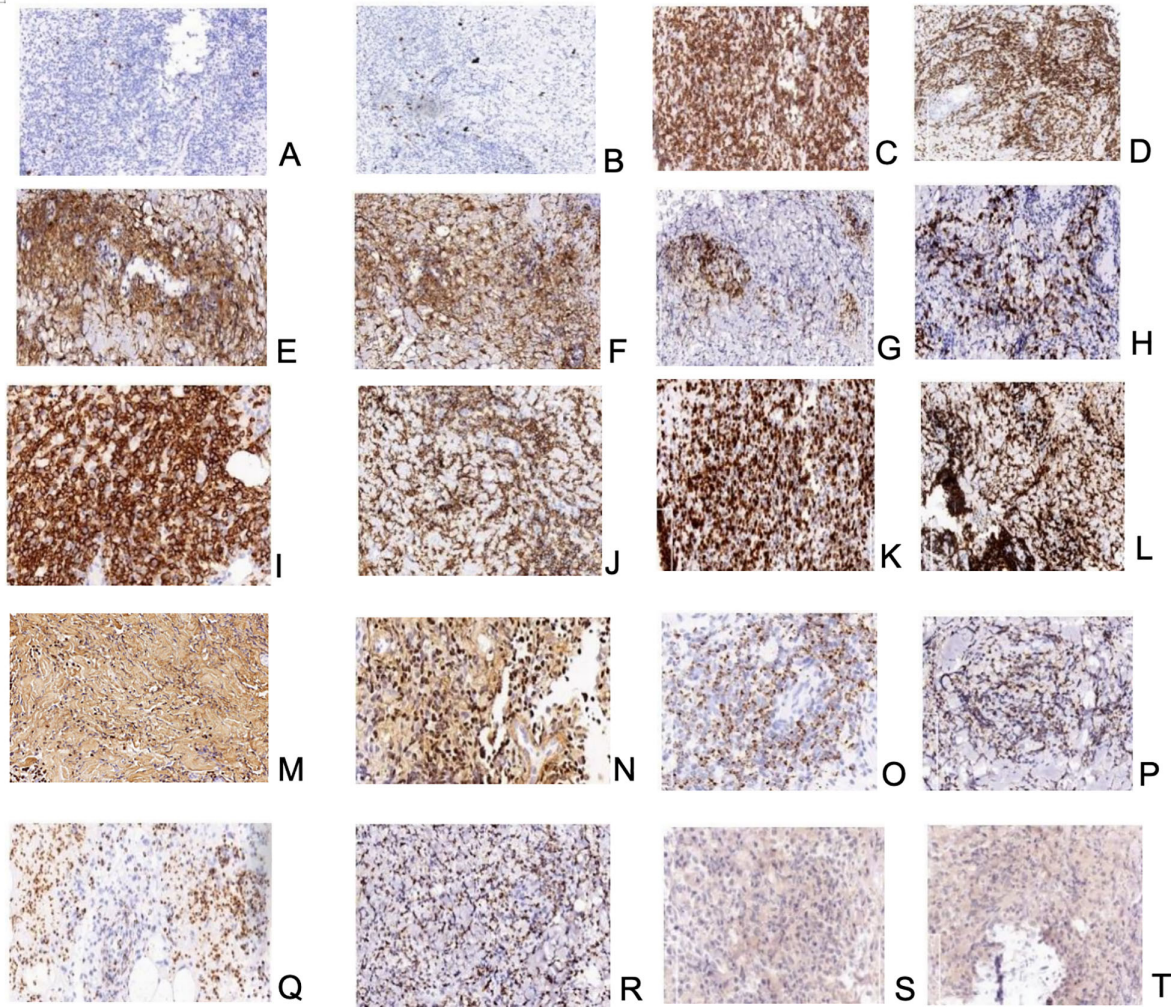
**(A, B)**-neoplastic NK/T-cells-positive reaction for CD20; biopsy of mass on the left upper extremity and the right upper extremity respectively; **(C, D)**–neoplastic NK/T-cells-positive reaction for CD3; biopsy of mass on the left upper extremity and the right upper extremity respectively; **(E, F)**–neoplastic NK/T-cells-positive reaction for CD4; biopsy of mass on the left upper extremity and the right upper extremity respectively; **(G, H)**–neoplastic NK/T-cells-positive reaction for CD8; biopsy of mass on the left upper extremity and the right upper extremity respectively; **(I, J)**–neoplastic NK/T-cells-positive reaction for CD56; biopsy of mass on the left upper extremity and the right upper extremity respectively; **(K, L)**– neoplastic NK/T-cells; high proliferative index – almost all cells showed expression of Ki67; biopsy of mass on the left upper extremity and the right upper extremity respectively; **(M, N)**–neoplastic NK/T-cells-positive reaction for EBER; biopsy of mass on the left upper extremity and the right upper extremity respectively; **(O, P)**–neoplastic NK/T-cells-positive reaction for Granzyme B; biopsy of mass on the left upper extremity and the right upper extremity respectively; **(Q, R)**–neoplastic NK/T-cells-positive reaction for TIA-1; biopsy of mass on the left upper extremity and the right upper extremity respectively; **(S, T)**–neoplastic NK/T-cells-positive reaction for EBNA2; biopsy of mass on the left upper extremity and the right upper extremity respectively.

In July 2021, 17 months after the transplantation, the patient reported a superficial mass in the left elbow joint with mild pain. The ultrasonography revealed a 2cm*2cm mass in the medial aspect of the left elbow joint. The biopsy was delayed due to the novel coronavirus epidemic. Twenty-one months after transplantation, the patient presented a 4.6*2cm subcutaneous mass on the right upper extremity. Puncture biopsies of bilateral upper extremity masses were performed, and similar characteristics were demonstrated. Pathological examination revealed that the mass was surrounded by the infiltration of abundant lymphocytes and heterogeneous cells, accompanied by granuloma formation and plentiful cellular necrosis. The immunohistochemical examination demonstrated the tumor cells expressed CD20, CD3, CD4, CD8, CD56, Ki67, Gr B, TIA-1, and EBNA2. They were also positive for EBV-encoded RNA (EBER) (**Figure 2**). EBV DNA was positive in PBMC at a low level and negative in plasma. A Positron Emission Tomography/computed tomography (PET/CT) indicated increased uptake of Fluoro-2-deoxy-D-glucose (FDG) in masses in both upper limbs, multiple lymph nodes, liver, spleen, truncal bones, colorectum, and multiple subcutaneous nodules. Consequently, the patient was treated with four courses of chemotherapy with L-GDP (L-Asparaginasum, Gemcitabine, Dexamethasone, Cisplatin) plus PD-1 inhibitor and was routinely evaluated by PET CT imaging at the end of treatment. The metabolic activity and volume of masses in both upper limbs and enlarged lymph nodes significantly decreased (with a Deauville score of 1-3), and no other abnormal lesions were revealed. CR was confirmed according to the Lugano efficacy evaluation criteria (8).

## Discussion

Lymphoid proliferations and lymphomas arising in post-transplantation display a bimodal distribution with an increase in incidence within one year of transplant and then another peak, which occurs around five years after transplant. Early-onset lymphoid proliferations and lymphomas arising in post-transplantation are mainly derived from polyclonal or monoclonal polymorphic B-cell proliferations, which occur within one year of transplantation and are frequently associated with EBV (9). In this case, the patient presented diffuse swelling and increased signal intensity of the spinal cord 11 months after transplantation and was clinically diagnosed with EBV-positive central nervous system (CNS) lymphoid proliferations and lymphomas arising in post-transplantation (10). Historically, only a minority of published cases of lymphoid proliferations and lymphomas arising in post-transplantation with neurological symptoms have been reported as case reports (11–13). Among them, involvement of the internal structure of the CNS occurs in approximately 5-30% of patients with lymphoid proliferations and lymphomas arising in post-transplantation (14). They often present with multiple supratentorial lesions in the periventricular regions (14). However, Beukelaar et al. reported that a rare lymphoid proliferations and lymphomas arising in post-transplantation case occurred in the ventral side of the spinal cord after HSCT (15). Another uncommon case of intraspinal lymphoid proliferations and lymphomas arising in post-transplantation involvement was reported in a pediatric patient who underwent renal transplantation (16). Our patient is a rare case of lymphoid proliferations and lymphomas arising in post-transplantation involving the spinal cord.

Due to the complexity of obtaining specimens from the CNS, multiple postoperative complications, and the rapid progression of CNS lymphoid proliferations and lymphomas arising in post-transplantation, most patients did not receive a histologically confirmed diagnosis or appropriate therapy during their lifetime. They commonly passed away within a year of receiving a transplant, with autopsy results ultimately confirming the diagnosis. According to previous reports, a combination of clinical presentation, imaging studies such as MRI, and the detection of EBV DNA in CSF can aid in the clinical diagnosis of CNS lymphoid proliferations and lymphomas arising in post-transplantation. Early initiation of treatment after clinical diagnosis of CNS lymphoid proliferations and lymphomas arising in post-transplantation can significantly improve the survival of patients, with an overall survival rate of up to a median of 47 months (17). However, standard prophylactic or therapeutic protocols for CNS lymphoid proliferations and lymphomas arising in post-transplantation are still under investigation (18). Current regimens for treating CNS lymphoid proliferations and lymphomas arising in post-transplantation include monotherapy with Rituximab (19), intrathecal injection of methotrexate (MTX) (20), high-dose MTX intravenous treatment (6), RIS combined with Rituximab and whole brain radiation therapy (WBRT) (21), and EBV-specific cytotoxic T lymphocytes (CTL) (22), all of which have demonstrated promising efficacy.

At 17 and 21 months post-transplantation, the patient presented with a mass on the bilateral upper extremities, along with enlarged multiple lymph nodes. However, the tissue biopsy was insufficient to confirm whether it was general NK/T cell lymphoma or NK/T cell lymphoma arising in post-transplantation. Misdiagnosis often occurs due to the similar pathological features shared by NK/T cell lymphoma arising in post-transplantation and common NK/T cell lymphoma (23). Early differentiation is especially crucial between NK/T cell lymphoma arising in post-transplantation and NK/T cell lymphoma, as it allows for the initiation of treatment. Notably, our patient had presented with generalized lymphadenopathy prior to treatment initiation. However, upon admission to our hospital, the enlarged lymph nodes had disappeared following chemotherapy with the DEP regimen, and no lymph node biopsy was performed for a definitive diagnosis. Therefore, it is reasonable to suspect the presence of occult lymphoma at the initial diagnosis, with a recurrence of occult lymphoma. However, NK/T cell lymphoma is predominantly extranodal (24), and patients with nodal involvement typically progress rapidly (25). The majority of patients have chromosomal abnormalities, such as del (6), del (8), and del (13), as well as frequent gene mutations, such as JAK3, STAT3, and STAT5b (26), which are not consistent with our patient’s clinical presentation at the time of initial treatment. Additionally, in NK/T cell lymphoma, increases in circulating EBV DNA are usually found due to viral DNA release from apoptosis of proliferating tumor cells. However, in this case, EBV DNA was positive in PBMCs at a low level and negative in plasma. On the other hand, multiple risk factors that increase the likelihood of developing lymphoid proliferations and lymphomas arising in post-transplantation have been elucidated, including the use of ATG prior to transplantation and immunosuppression following HSCT, elderly donor (51 years), difference of EBV serological status between donor and recipient, and haplo-identical HSCT (27). Pathology indicated that the tumor cells predominantly exhibited an EBV latency type III (LMP1-positive, EBNA2-positive, EBER-positive), mainly expressed in immunodeficient patients (28). Wang S.H. et al. reported a patient who underwent HSCT for cutaneous NK/T cell lymphoma and developed hepatosplenomegaly and cervical lymphadenopathy two months after transplantation. The patient was ultimately diagnosed with EBV-positive lymphoma arising in post-transplantation, although recurrence of NK/T cell lymphoma was suspected initially (29). Even though the pathological presentations of lymphoid proliferations and lymphomas arising in post-transplantation with cutaneous involvement are commonly characterized by polymorphic or monomorphic B cell and plasma cell subtypes (30–32), the NK/T cell lymphoid proliferations and lymphomas arising in post-transplantation primarily manifesting as subcutaneous nodules have also been reported (9, 33, 34). The majority of them are usually present late after transplantation and are EBV-negative (27). Nonetheless, approximately 15% of NK/T cell lymphoid proliferations and lymphomas arising in post-transplantation occur in the early post-transplant stage (5), and about 40% of these patients are EBV-positive. Based on the evidence presented, the final diagnosis of the subcutaneous mass was established as NK/T cell lymphoma arising in post-transplantation.

RIS has been the cornerstone of first-line treatment for lymphoma arising in post-transplantation (35), and it is often used in combination with chemotherapy, radiotherapy, surgery, adoptive T-cell therapy, and antiviral and immunological agents. The combined treatment has become the mainstream for NK/T cell lymphoma arising in post-transplantation, and the 5-year survival rate has risen to 60% (34). NK/T cell lymphoma is an aggressive disease with a poor response to therapy and a high risk of replase, resulting in a poor long-term prognosis. The overall 5-year survival rate is approximately 10 to 40%, with the median survival being 15 months (36–38). Patients with extracutaneous involvement show shorter median survival (39). On the contrary, as of the last follow-up in March 2023, our patient maintained CR without any evidence of disease recurrence. The satisfactory treatment efficacy of the patient further supported the diagnosis of NK/T cell lymphoma arising in post-transplantation.

EBV infection status is a significant factor associated with the development of lymphoid proliferations and lymphomas arising in post-transplantation (40). Unlike post-transplant B cell lymphoid proliferations and lymphomas arising in post-transplantation, the role of EBV in EBV-positive NK/T cell lymphoid proliferations and lymphomas arising in post-transplantation is still unclear. Magro et al. suggest that regulatory T cells can undergo tumorigenic transformation under conditions of immunosuppression. EBV-infected B cells, serving as a continuous antigenic stimulus, may induce an excessive immune response in T cells, leading to the development of EBV-positive NK/T cell lymphoid proliferations and lymphomas arising in post-transplantation (41). The incidence of lymphoid proliferations and lymphomas arising in post-transplantation has significantly increased over the last two decades due to various factors, including an increasing number of HSCT, older donors and recipients, the use of novel immunosuppressive agents, and the introduction of unrelated donors (42). Despite significant improvements in supportive strategies following HSCT in recent years, many problems still need to be better controlled. Monitoring EBV DNA allows for early recognition of impending lymphoid proliferations and lymphomas arising in post-transplantation, thus providing a basis for timely treatment initiation (43). Notably, only 30% of case reports showed positive results for EBV DNA in the CSF of patients with CNS lymphoid proliferations and lymphomas arising in post-transplantation (44); for post-transplant patients who present with CNS symptoms, peripheral blood EBV DNA monitoring does not meet clinical needs, making combined imaging examinations necessary for monitoring CNS lymphoid proliferations and lymphomas arising in post-transplantation. Furthermore, there needs to be more standardization across institutions in the detection methods and the sample types used for EBV DNA surveillance. The management of lymphoid proliferations and lymphomas arising in post-transplantation also needs a common consensus around the EBV DNA threshold for preemptive therapy. The standard RIS regimens that allow for the elimination of lymphoid proliferations and lymphomas arising in post-transplantation while maintaining the level of immunosuppression to prevent graft rejection and GVHD have yet to be elucidated. Additionally, the optimal dosage of Rituximab in first-line treatment regimens warrants further investigation (45).

## Conclusion

Conclusively, lymphoid proliferations and lymphomas arising in post-transplantation with predominantly intraspinal involvement is a rare disorder that is difficult to diagnose definitively and has a dismal clinical prognosis. This case report serves as a reminder to clinicians to maintain a high index of suspicion for lymphoid proliferations and lymphomas arising in post-transplantation with spinal cord involvement when neurological complications arise after HSCT. It also highlights the importance of long-term imaging and CSF monitoring in post-transplant patients. Early diagnosis is crucial for disease management and improvement in prognosis due to the differences in pathomechanisms and prognosis between NK/T cell lymphoma arising in post-transplantation and general NK/T cell lymphoma. This article further explains the current treatment modalities and clinical shortcomings of lymphoid proliferations and lymphomas arising in post-transplantation, providing more references to enhance the knowledge of clinicians and pathologists on the disease and pointing out the direction for future exploration.

## Data Availability

The original contributions presented in the study are included in the article/supplementary material. Further inquiries can be directed to the corresponding authors.
